# Cylindrical Polyurethane Scaffold Fabricated Using the Phase Inversion Method: Influence of Process Parameters on Scaffolds’ Morphology and Mechanical Properties

**DOI:** 10.3390/ma14112977

**Published:** 2021-05-31

**Authors:** Aleksandra Kuźmińska, Dominika Kwarta, Tomasz Ciach, Beata A. Butruk-Raszeja

**Affiliations:** 1 Biomedical Engineering Laboratory, Faculty of Chemical and Process Engineering, Warsaw University of Technology, Warynskiego 1, 00-645 Warsaw, Poland; dominika.kwarta.stud@pw.edu.pl (D.K.); Tomasz.Ciach@pw.edu.pl (T.C.); Beata.Raszeja@pw.edu.pl (B.A.B.-R.); 2 Centre for Advanced Materials and Technologies CEZAMAT, Warsaw University of Technology, Poleczki 19, 02-822 Warsaw, Poland

**Keywords:** phase inversion technique, tissue scaffold, polyurethane, surface porous

## Abstract

This work presents a method of obtaining cylindrical polymer structures with a given diameter (approx. 5 mm) using the phase inversion technique. As part of the work, the influence of process parameters (polymer hardness, polymer solution concentration, the composition of the non-solvent solution, process time) on the scaffolds’ morphology was investigated. Additionally, the influence of the addition of porogen on the scaffold’s mechanical properties was analyzed. It has been shown that the use of a 20% polymer solution of medium hardness (ChronoFlex C45D) and carrying out the process for 24 h in 0:100 water/ethanol leads to the achievement of repeatable structures with adequate flexibility. Among the three types of porogens tested (NaCl, hexane, polyvinyl alcohol), the most favorable results were obtained for 10% polyvinyl alcohol (PVA). The addition of PVA increases the range of pore diameters and the value of the mean pore diameter (9.6 ± 3.2 vs. 15.2 ± 6.4) while reducing the elasticity of the structure (Young modulus = 3.6 ± 1.5 MPa vs. 9.7 ± 4.3 MPa).

## 1. Introduction

Research is continuously carried out to create the ideal tissue scaffold. This is particularly important in the investigation of vascular prostheses [1]. Vascular grafts larger than 6 mm in diameter are widely available [2]. The production of artificial vascular grafts of smaller diameter (diameter less than 6 mm [3])—in contrast to those of larger sizes—is associated with many difficulties [4]. For this reason, it is necessary to search for new techniques for the production of small vascular prostheses. Vascular prostheses with small diameters, like those with larger diameters, must have appropriate parameters. In addition to bio- and hemocompatibility, one of these criteria is a surface with proper pore sizes [5,6,7,8]. A surface pore size adapted to the cell size will facilitate the process of their adhesion to a material and further proliferation [9,10]. Moreover, a well-chosen pore size will accelerate and improve the prosthesis’s integration with the surrounding tissues [11]. Appropriate surface pores additionally will allow cells to exchange nutrients, a limiting factor in small diameter prostheses [5,12]. This is a crucial aspect since the endothelialization of the material is the most effective method to prevent thrombosis [13].

Vascular prostheses with large diameters are usually made of polymers, such as polyethylene terephthalate (PET) and expandable polytetrafluoroethylene (ePTFE) [14,15]. However, in the case of vascular prostheses with smaller diameters, these polymers’ use is problematic due to clot formation, clogging the prosthesis’s lumen. Therefore, for the preparation of prostheses of small diameter, polyurethanes (PU) are increasingly used [16,17,18]. PUs are already used in cardiological devices, such as heart valves or vascular stents [19,20]. Apart from acceptable bio- and hemocompatibility [7,21], polyurethanes are characterized by good mechanical strength [22]. The biocompatibility of numerous PUs has been studied In Vitro and in vivo for many applications [23]. They also maintain a good cell adhesion and rate of cell proliferation on their surfaces [24].

Many different methods can be used to produce vascular prostheses, e.g., electrospinning, solution blow spinning, phase inversion, and solvent casting/particulate leaching [5,7,25,26]. Phase inversion is a simple method that does not require expensive and complicated equipment. Phase inversion is a process during which a third component (polymer nonsolvent) is introduced into the two-component polymer-solvent system. During the process, the polymer is turned from a solution into a solid state. The basis of the process is the difference in the polymer solubility between the solvent and nonsolvent. As a result, phase separation occurs, and two phases are created—rich and poor—in a polymer. This process is caused by the diffusion of solvent from the polymer solution to the precipitation bath, and the nonsolvent from the precipitation bath to the polymer solution [27,28,29]. Pores are formed due to the loss of solvent and nonsolvent [30]. Changing the process parameters allows obtaining surfaces with different morphologies. The porosity can be increased by adding a porogen [31]. In the case of vascular prostheses, in addition to an appropriate surface morphology, a crucial aspect is the good mechanical properties of the scaffold is needed to maintain the vessel’s structural integrity under high pressure and flow [32].

The study aimed to assess the effect of selected process parameters on the morphology of polyurethane structures that can potentially be used as a small diameter vascular prosthesis. The appropriate morphology of such a scaffold and its mechanical properties are crucial, as outlined above. ChronoFlex polyurethane was used in the presented study. It is often used in cardiac implant investigations [33,34,35,36,37], most often to produce vascular prostheses by electrospinning [38,39,40]. Cylindrical structures were obtained using the phase inversion process. Various types of polyurethanes (differing in flexibility), different polymers concentrations, different nonsolvents, and different process times were investigated. Moreover, the influence of porogen addition on scaffold morphology and elasticity was studied. The cytotoxicity of the obtained scaffolds was also analyzed.

## 2. Results

### 2.1. Selection of Process Parameters

Cylindrical vascular prostheses with an internal diameter of approx. 5 mm were fabricated using the phase inversion technique described below. At each stage of the study, the main focus of the analysis was put on the inner surface of the obtained materials. The expected features were a structure devoid of artifacts with a microporous surface and high elasticity. The surface pores should be uniform, of similar sizes. Furthermore, the surface should be free from irregularities as this could promote platelet adhesion and clot formation after contact with human blood. Three types of PUs (C75A, C45D, C75D), differing on the Shore scale, were examined. According to this scale, C75A polymer is a soft polymer the C45D and C75D polymers hard ones.

#### 2.1.1. Composition of Nonsolvent Solution

Table 1 provides a description of the structures obtained for different compositions of the nonsolvent solution for the three tested PUs (C75D, C75A, C45D). The concentration of the polymer solution (20% *w*/*v*) and the process time (24 h) were constant.

For the material made of C75A, for low ethanol concentrations in the nonsolvent, the internal structure was irregular, full of artifacts, and large macropores. During drying of the samples, there was a problem with sticking to the walls. The walls of the scaffolds were so thin that they collapsed. A satisfactory outer surface was obtained for the highest concentration of ethanol as a nonsolvent. With a higher ethanol content in the nonsolvent, more stable cylindrical structures were obtained, the walls of which did not collapse. At 100:0 water/EtOH, the inner surface was nonuniformly porous—some areas remained featureless—fine spherical pores could be observed on the rest. The internal surface of the structure obtained at 80:20 water/EtOH was very similar to that obtained at 100:0 water/EtOH, though more micropores could be observed. For 60:40 water/EtOH, the inner surface was uniformly porous with spherical surface micropores. At 40:60 water/EtOH, it was not possible to analyze the inner surface. During drying, the material’s walls collapsed and stuck together, for this reason, an SEM photo of the outer surface is provided. At 20:80 water/EtOH, the internal surface was heterogeneously porous; however, an increase in the number of pores can be seen. At 0:100 water/EtOH, the inner surface was also porous, with spherical pores of varying sizes. In some small areas, the structure resembled a sponge.

Similar results were obtained for C45D; however, a cylindrical structure was obtained from all tested nonsolvent combinations. With a 100:0 water/EtOH mixture, many artifacts, including spherical polymer particles, could be observed on the inner surface. With 80:20 water/EtOH, a similar surface to the 100:0 water/EtOH variant was obtained, except that individual surface micropores in the groups could be seen. At 60:40 water/EtOH, part of the inner surface remained featureless and areas with more pores and similar sizes appeared. Here, surface artifacts could also be observed. With the next nonsolvent tested, 40:60 water/EtOH, there were more pores of similar sizes on the inner surface, and smaller areas remained featureless. For 80:20 water/EtOH, the surface was fairly uniformly porous and there were no areas without visible pores; however, artifacts were still visible on the surface. For 0:100 water/EtOH, the inner surface was homogeneously porous. The pores were round and similar in shape, and the surface was smooth with artifacts.

In the case of PU C75D, it was possible to obtain cylindrical scaffolds for all tested nonsolvents. The higher the ethanol concentration in the nonsolvent solution was, the smoother the outer surface and the more porous the inner surface was. For a 100:0 water/EtOH mixture, the inner surface was smooth but with very irregularly shaped open pores. At 80:20 water/EtOH, the surface porosity increased slightly and the pores were different sizes. Similarly, for 60:40% water/EtOH, more spherical and regular pores could be observed on the inner surface; however, they were still relatively small. The material surface obtained for 40:60 water/EtOH had a sponge-like structure. The pores were larger but had very heterogeneous shapes and sizes. For the 20:80 water/EtOH mixture, the inner surface had a sponge-like structure on a larger surface area; more surface pores appeared. In the case of the highest ethanol content in the nonsolvent, 0:100 water/EtOH, the inner surface was uniformly porous and spherical pores of similar sizes could be observed on the surface.

**Figure 1:**
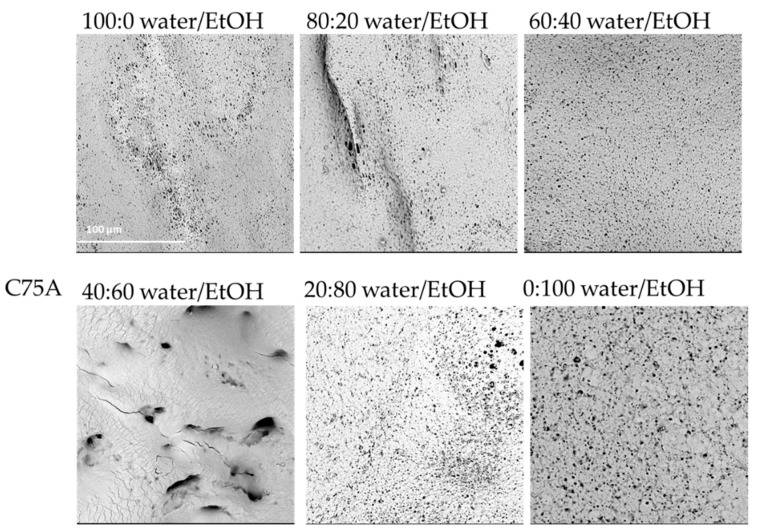
Morphology of internal surface of cylindrical scaffolds obtained from ChronoFlex C75A for different nonsolvent compositions. For 40:60 water/EtOH only the outer surface was observed; scale bar: 100 μm.

**Figure 2:**
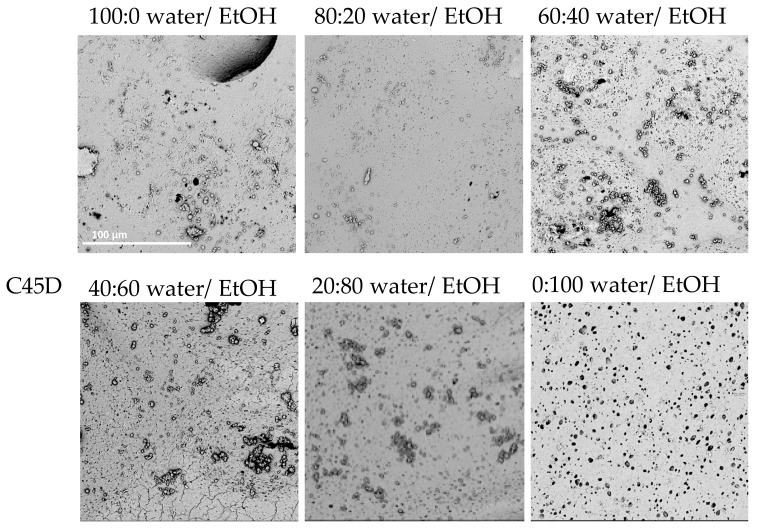
Morphology of internal surface of cylindrical scaffolds obtained from ChronoFlex C45D for different nonsolvent compositions; scale bar: 100 μm.

**Figure 3:**
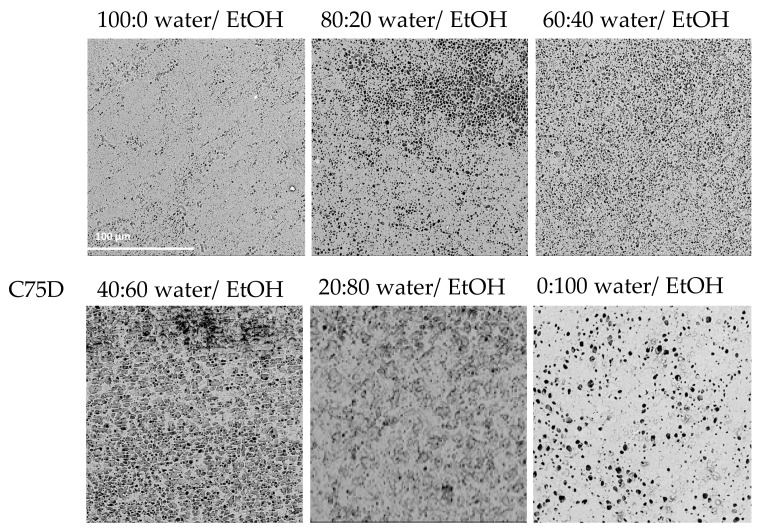
Morphology of internal surface of cylindrical scaffolds obtained from ChronoFlex C75D for different nonsolvent compositions; scale bar: 100 μm.

Figure 1, Figure 2 and Figure 3 present SEM photos for all tested combinations of nonsolvents, respectively, C75A (Figure 1), C45D (Figure 2), and C75D (Figure 3).

For all three tested polyurethanes, it can be seen that the higher the ethanol content in the nonsolvent solution was, the more uniformly porous the inner surface and the smoother the outer surface was. The most favorable surfaces were obtained for the nonsolvents with the highest content of EtOH (0:100 water/EtOH). They had evenly porous surfaces with pores of similar diameters. The manually assessed elasticity was the best in this nonsolvent variant. Moreover, with lower EtOH contents, the scaffold walls tended to collapse during drying, especially for the more elastic PUs, i.e., C75A and 45D.

#### 2.1.2. Concentration of Polymer Solution

Table 2 shows a symbolic description of the structures obtained for different PU concentrations. Analyses were performed for three types of PUs (C75D, C75A, C45D). The composition of the nonsolvent solution (0:100 water/EtOH) and the process time (24 h) were constant.

With PU C75A, it was possible to obtain cylindrical materials from all PU concentrations. The inner surface of 10% PU was slightly porous and some parts remained featureless. For the inner surface of 15% PU, numerous artifacts could be seen and the surface resembled a sponge. The surface of the 20% PU structure also resembled a sponge, but was not as porous. The scaffold surface with 25% PU was similar to that obtained with 20%, but was more porous. There were also visible artifacts on the surface.

In the case of C45D, for 10% PU, the scaffold walls were very thin, which was problematic during drying. The inner surface was full of artifacts and was very heterogeneous. For 15% PU, it was not possible to analyze the inner surface due to the fact that, during drying, the walls stuck together. The 20% PU material was homogeneously porous with spherical pores. When 25% PU was used, the surface was more porous, rather heterogeneous, and more surface irregularities appeared.

In the case of C75D, cylindrical scaffolds were obtained for all concentrations of PU. The 10% PU scaffold’s inner surface was heterogeneously porous—the pores were spherical and of various sizes. By increasing the polymer concentration to 15% PU, a homogeneous surface structure was obtained, but the pores were relatively small. Using 20% PU, the inner surface was also uniformly porous, with surface pores larger than those of 15% PU. The structure obtained from a 25% solution had an inner surface which was highly porous, resembling a sponge.

**Figure 4:**
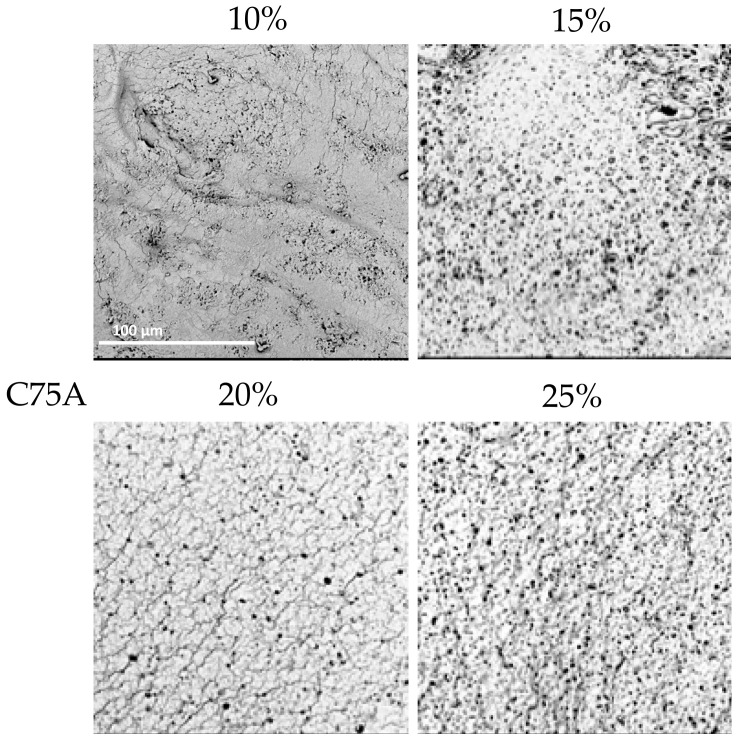
Morphology of internal surface of cylindrical scaffolds obtained from ChronoFlex C75A at different concentrations of polymer solution; scale bar: 100 μm.

**Figure 5:**
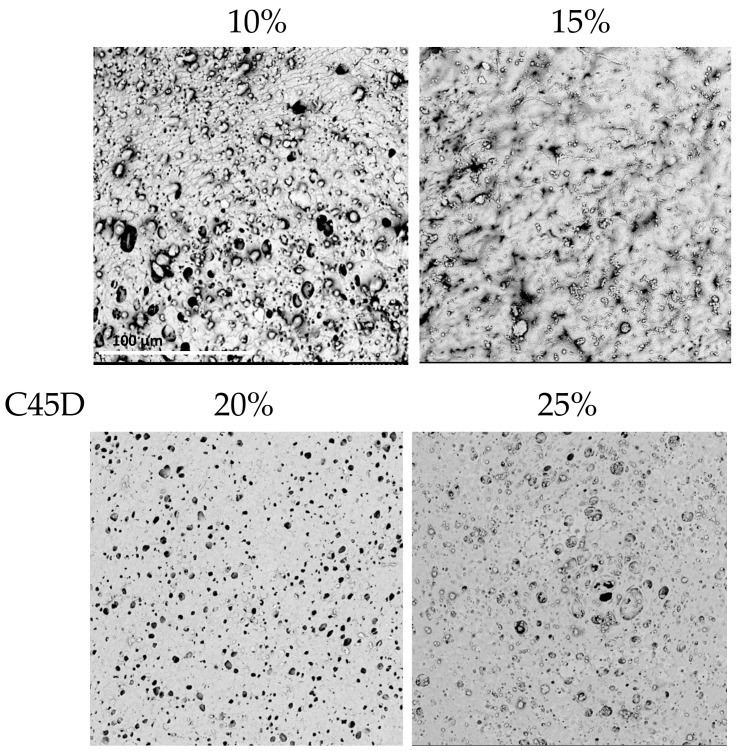
Morphology of internal surface of cylindrical scaffolds obtained from ChronoFlex C45D at different concentrations of polymer solution. For 15% PU only the outer surface was observed; scale bar: 100 μm.

**Figure 6:**
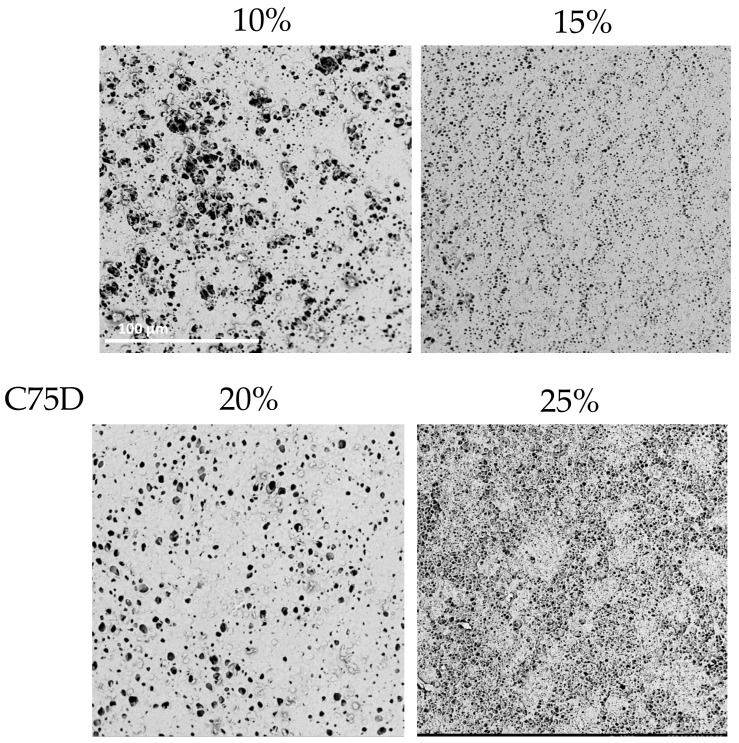
Morphology of internal surface of cylindrical scaffolds obtained from ChronoFlex C75D at different concentrations of polymer solution; scale bar: 100 μm.

In conclusion, for all analyzed types of PUs, the structures obtained from the lower polymer concentrations had thin walls; no thicker structures could be obtained because the polymer solution dripped from the matrix. Moreover, with a lower PU concentration, the walls tended to collapse during drying. Furthermore, structures obtained with 25% PU were stiffer (determined by a manual evaluation) than those with 20% PU. The highest used PU concentration gave materials with thick walls and a lower elasticity, which was assessed manually. This was due to the high density of the polymer solution. SEM analyses (Figure 4, Figure 5 and Figure 6) indicated that the 20% PU concentration was the most favorable variant as the surface was evenly porous and had pores of similar sizes. Examination of the SEM images showed that the obtained structures showed a very low presence of artifacts and have a number of sought-after features—homogeneity and microporosity—for the surface.

#### 2.1.3. Time of the Process

Table 3 presents a symbolic description of the structures obtained for different process times. Analyses were performed for three types of PUs (C75D, C75A, C45D). The composition of the nonsolvent solution was constant (0:100 water/EtOH) and the concentration of the polymer solution was also constant (20% *v*/*w*).

Cylindrical structures were obtained for all analyzed times. In the case of C75A, no porous inner surface was obtained after 10 min. In the next tested period, 2 h, single pores were observed. Only after 24 h was a porous homogeneous inner surface obtained. In the case of C45D, along with a longer process time, a more porous and uniform internal surface was obtained. For C75D, a porous structure was obtained after 10 min—the inner surface was heterogeneous, resembling a sponge in some places. After 2 h, the surface was more uniformly porous. However, for both time variants, the outer surface was strongly folded. For the longest tested time, the most homogeneous porous inner surface was achieved.

**Figure 7:**
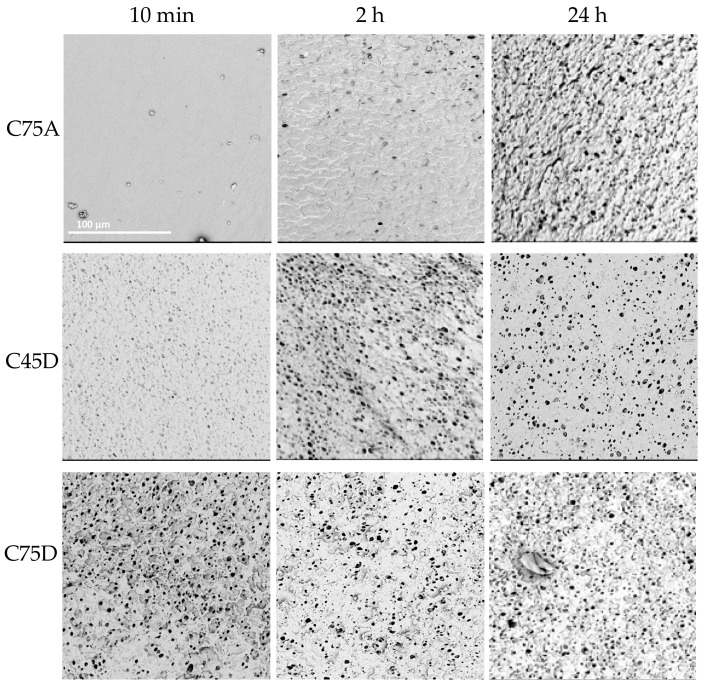
Morphology of internal surface of cylindrical scaffolds obtained from ChronoFlex C75D, C75A and C45D for different process times; scale bar: 100 μm.

The obtained results (Figure 7) showed that the most favorable morphology was when the process time was 24 h. The received structures were devoid of defects (interruption of the wall), as well as with the inner surfaces having the desired features.

The obtained analyses led to selecting the following process parameters: 20% PU concentration, 0:100 water/EtOH as a nonsolvent, and a process time of 24 h for all three types of PUs. The materials produced with this set of process parameters best met the above-defined criteria—a uniformly porous inner surface with pores of similar size, and without a large number of artifacts or irregularities on the inner surface. The outer surface was somewhat porous and not folded too much.

MTT assay results showed a cell viability of about 90% (relative to negative control) for all materials (no statistically significant differences, *p* < 0.05) (Table 4). The obtained materials (from all three polymers) could be considered nontoxic [41]. In Table 4, the mechanical properties of materials from the three tested polymers are presented; differences between the test parameters are statistically significant (*p* < 0.05). Materials made of PU C75A were thin and had uneven wall thicknesses; moreover, they were too flexible. PU C75A materials had the lowest Young’s modulus from the studied polymers and the highest elongation at break. PU C45D materials showed less flexibility than PU C75A, but this was rated as a benefit. Moreover, it was possible to produce even-walled structures without damaging the surface. PU C75D materials were very stiff and had the highest Young’s modulus. Furthermore, they crumbled after a longer storage time.

Thus, based on the analyses of the surface morphology and the mechanical properties, PU C45D was selected for further research. The process parameters were 20% polymer concentration, 0:100 water/EtOH as a nonsolvent solution, and a process time of 24 h.

#### 2.1.4. Selection of Porogen

The 24-h variant of 20% PU C45D, 0:100 water/EtOH was selected to analyze the influence of porogen on the obtained structure. The addition of three substances (NaCl, PVA, hexane) in two concentrations (5%, 10%) was examined. Table 5 presents a symbolic description of the obtained structures. It was possible to obtain cylindrical structures for all tested porogens. The expected features were a homogeneously porous surface with surface pores of similar sizes (above 10 μm). The surface should not be folded too much or have additional cracks. Moreover, the outer surface should not be too porous.

**Figure 8:**
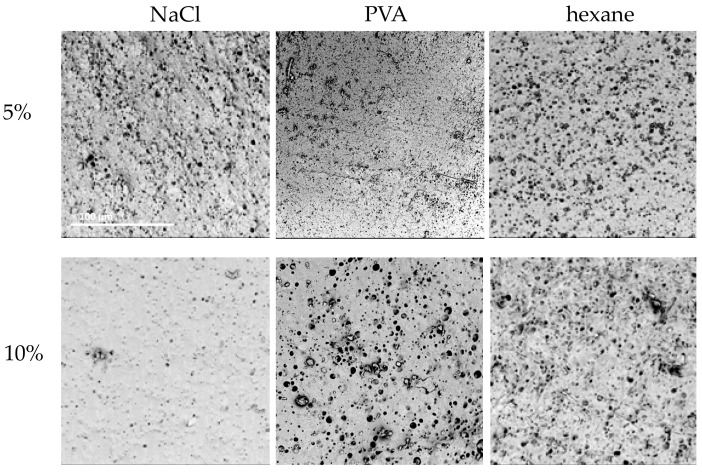
Morphology of internal surface of cylindrical scaffolds obtained from ChronoFlex C45D (20%, 0:100 water/EtOH, 24 h) with the addition of different porogens; scale bar: 100 μm.

Figure 8 presents SEM images of the obtained materials. Smaller concentrations of porogens made the surface more folded than porous. Higher concentrations resulted in surfaces with larger pores. With the addition of 5% NaCl, the outer surface was heavily wrinkled with irregular surface pores. Some roughness can be observed on the inner surface and the surface pores are heterogeneous.

When using 10% NaCl, the outer surface was highly porous in some places and relatively smooth with single surface pores in others. The inner surface was more homogeneous compared to the materials obtained with 5% NaCl, but no increase in the number of pores was noted. The addition of 5% PVA did not significantly change the outer surface compared to the materials obtained without this porogen. There were single-surface artifacts. More heterogeneous pores could be observed on the inner surface, yet the pores were relatively small. When using 10% PVA, surface pores and slight ripples appeared on the outer surface. The inner surface was uniformly porous—there were individual, larger and smaller pores, but most of them were of similar sizes. The use of 5% hexane caused the outer surface to be significantly folded; there were single-surface pores. In contrast, more surface pores could be observed on the inner surface and there were no surface artifacts. With the addition of 10% hexane, the outer surface was not as wrinkled as with 5%, but more surface pores could be observed. The inner surface had more inhomogeneous porosity. Considering the above results, 10% PVA was chosen as the most effective porogen as the resulting material best met the expectations.

### 2.2. Influence of Porogen Addition on Physical and Mechanical Properties

**Figure 9:**
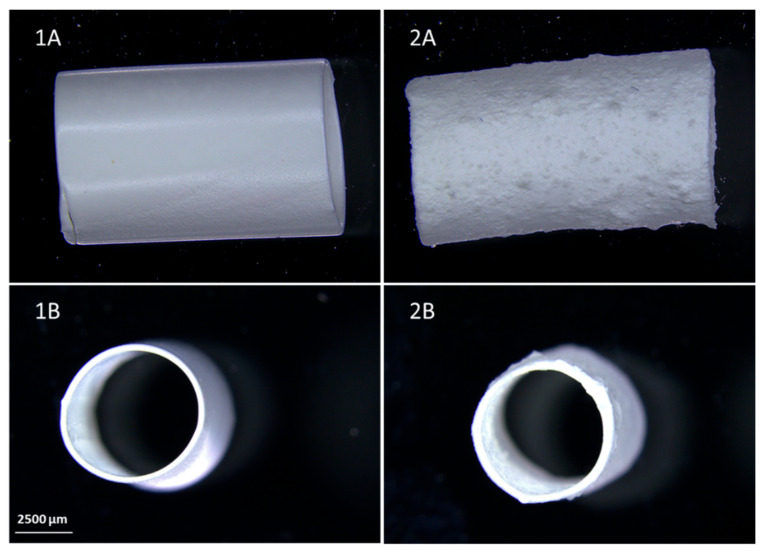
Comparison of the macroscopic images of samples (**A**) and their cross-sections (**B**), PU C45D (**1**), PU C45D_10%PVA (**2**); scale bar: 2500 μm.

Figure 9 shows pictures of PU C45D and PU C45D_10% PVA prostheses. It can be seen that the outer surface of PU C45D is smooth, while PU C45D_10% PVA is much rougher. This is the result of the addition of the porogen to the polymer solution and its irregular distribution on the stainless-steel matrix, despite its equal distribution in the PU solution.

**Figure 10:**
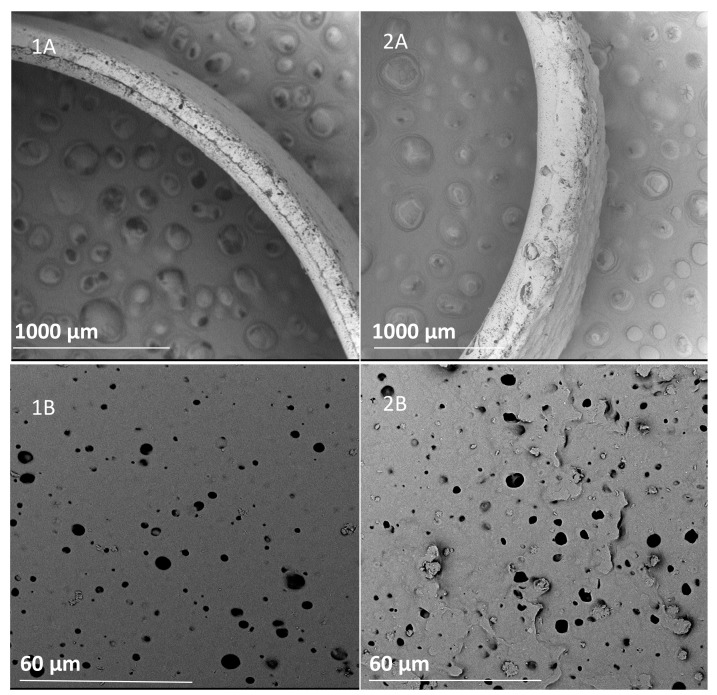
Comparison of representative SEM images of cross-sections (**A**) and sample surface (**B**), PU C45D (**1**), and PU C45D_10% PVA (**2**).

As shown in the SEM images in Figure 10, the PVA changed the materials’ internal structures. From the cross-section of the scaffolds, more pores, not only surface ones, can be seen; macropores are also present. 

Table 6 presents basic physical parameters for PU C45D and PU C45D_10% PVA. The wall thicknesses were 202 ± 19 μm and 278 ± 78 μm for the materials, without and with the porogen, respectively. The higher SD value was due to the addition of porogen and the deterioration of the structure. The wall thicknesses for 10% PVA materials are significantly thicker due to the addition of PVA (*p* < 0.05).

**Figure 11:**
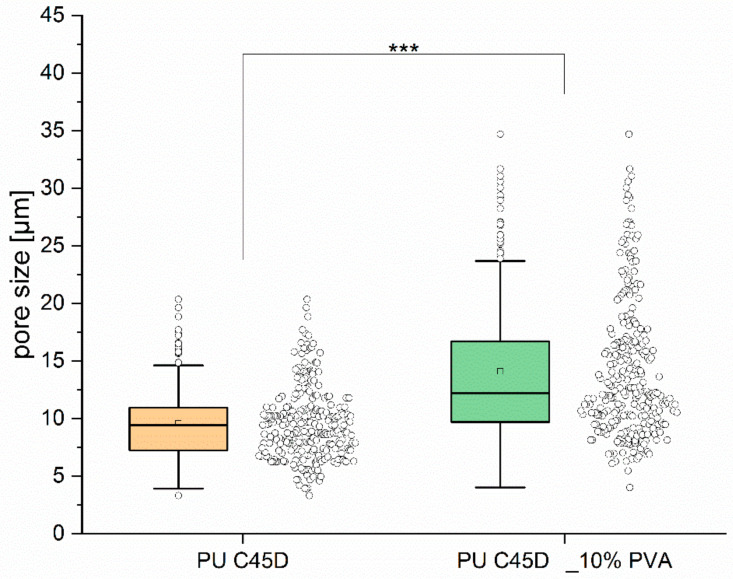
Pore size distribution for PU C45D and PU C45D_10% PVA, ***p < 0.001.

As can be seen from the results in Table 6, the addition of porogen significantly (*p* < 0.05) increased the average surface pore diameter (from 9.6 ± 3.2 μm to 15.2 ± 6.4 μm). Moreover, the maximum surface pore diameter for PU C45D_10% PVA was twice as large as that for scaffolds without porogen (41.2 μm vs. 20.4 μm, respectively). Furthermore, PVA addition statistically significantly (*p* < 0.05) increased sample porosity by 9% (from 59% ± 2% to 68% ± 3%). Thus, the intended goal of increasing surface porosity was achieved. Figure 11 presents the pore size distribution for both material variants. In the case of materials without porogen, the pore size distribution is narrower (3–10 μm) compared to PU C45D_10% PVA (5–40 μm). In addition, there are more medium-sized pores (of about 10 μm). The addition of porogen significantly (*p* < 0.001) extends the range of pore diameters. Moreover, PVA addition statistically significantly increased the sample porosity by 9% (from 59% ± 2% to 68% ± 3%). The addition of porogen decreased the cell viability (from 92 ± 4% to 86 ± 9%), yet this was not a significant difference. Both materials are not toxic.

The addition of porogen changed the mechanical properties of the materials. The average Young’s modulus for PU C45D was 3.6 ± 1.5 MPa. The value for C45D_10% PVA was higher (9.7 ± 4.3 MPa), which means that after the addition of porogen the prostheses were much stiffer—a higher force was required for the deformation of such materials (not statistically significant difference, *p* < 0.05). The tensile strength of the material with porogen was two-fold lower compared to the material without PVA addition (statistically significant difference, *p* < 0.05). Moreover, the elongation at break decreased with porogen addition (statistically significant difference, *p* < 0.05).

## 3. Discussion

Vascular prostheses can be obtained using various techniques. Fibrous materials are the most popular materials studied so far for use as vascular prostheses [42,43]. Fibrous vascular prostheses are fabricated to closely match natural blood vessel behavior. Electrospinning [44,45,46,47] and solution blow spinning [48,49] are most often used; these methods provide scaffolds with different fiber diameters and a high porosity, which are very good for substrates in terms of cell growth. Moreover, such materials are usually characterized by their high flexibility and breaking strength [43]. However, special equipment is needed to manufacture fibrous scaffolds. In addition, selecting the most favorable parameters, such as flow, voltage, etc., can be very labor intensive and the process depends on many variables [50]. The need to use highly volatile solvents is also a major limitation of the process. For some polymers, such as ChronoFlex, the selection of such a solvent is difficult. Our group also conducted research on the production of fibrous structures from ChronoFlex using the solution blow spinning (SBS) technique [51,52]. The only solvent that made it possible to obtain fibers using this method was 1,1,1,3,3,3-hexafluoro-2-propanol (HFIP). The disadvantage of this solvent is its high cost; which forced us to look for alternative methods of obtaining cylindrical structures.

The phase inversion method is an interesting alternative; however, it also has disadvantages—the complexity of the process and possible uneven wall thickness—which can be counteracted using standardized methods, e.g., the extrusion process [53] or thermal-induced phase inversion [54,55]. In addition, the differences between the various phase inversion techniques make it possible to achieve different microporosities [3,56]. As already mentioned, the phase inversion process depends on many parameters. The use of polyurethane and phase inversion methods to produce scaffolds that may be a base for vascular prostheses allows the obtaining of a material that meets the criteria for a suitable scaffold, i.e., good mechanical properties, biocompatibility, ease of manufacture, and appropriate morphology [1,57].

The primary purpose of the present research was to complement fundamental knowledge on the production of cylindrical polyurethane structures with surface pores using the phase inversion method. This allows investigating the effect of individual process parameters on the distribution and size of surface pores. The number of papers describing the impacts of various process parameters on pore distributions in the resulting structure is not sufficient, especially in terms of using ChronoFlex. In addition, an important aspect of the proposed study is the evaluation of material toxicity. The goal of this part of the work was to create structures that could be used as prostheses of blood vessels, so they should have a specific internal diameter, wall thickness that allows the cylindrical structure to be also maintained under dynamic conditions as well as appropriate flexibility and porosity. The inner surface of such materials deserves special attention. The importance of surface porosity in artificial blood vessels has been emphasized for many years [58].

Our aim was to create a porous structure that would promote the restoration of the endothelial structure. For the prosthesis’ inner surface, the minimal recommended pore size was 10 µm, which will allow the formation of a monolayer of endothelial cells [59]. Restoration of the endothelium will increase the hemocompatibility of such a surface and reduce the risk of blood clotting [10,60]. The outer side should also be uniform; cracks and tears are undesirable and indicate a structure’s poor mechanical properties.

ChronoFlex is often used in studies on materials contacting with blood [19,61,62]. The presented research aimed to evaluate the selected parameters’ influence on polyurethane cylindrical scaffolds’ morphology. Khorasani and Shorgashti presented similar studies on the influence of parameters on the morphology of obtained flat surfaces. They investigated the effects of polyurethane concentration, process temperature and composition nonsolvent solution [63]. Here, various types of ChronoFlexes at several concentrations, various nonsolvents and with different process times were tested. First, the influence of the nonsolvent composition on the morphology of the maintained structure was investigated. Six solutions that differed in water:ethanol ratios were tested. A comparison of all samples showed that 0:100 water/EtOH was the most preferred choice of nonsolvent. Selected variants had a microporous structure. In addition, the materials were free from cracks, and the artifacts that were found occurred in relatively small amounts. The flexibility of the samples, assessed manually, was also the best in these samples. It could be noticed that the higher the ethanol concentration in the nonsolvent solution was, the better the obtained structure was, which met the assumed criteria. The result obtained corresponded to that of Khorasani and Shorgashti [63]; who studied water and various alcohols as nonsolvents. They observed that when an alcohol solution replaces water in nonsolvent, macrovoids are reduced.

Another analyzed process parameter was polymer concentration. At low analyzed polymer concentrations, the system was a low viscosity liquid [56]. Lower concentrations (10% and 15%) were inadequate as the polymer dripped from the matrix, and thin walls were obtained with very uneven thicknesses. At 25% PU, the inner surface had more surface porosity; however, the outer surface material was also more porous. A 25% PU solution was too dense, so material walls were uneven, and the materials were also stiff. Therefore, we decided to use 20% PU. The literature reports that, as a polymer concentration increases, surface porosity decreases [63,64]. However, in the experiments presented here, a smaller surface porosity was observed on the surface of materials made using lower tested concentrations. The differences probably resulted from the nonsolvent used, which proves how strongly it affected the resulting structure.

With the longest tested process time, i.e., 24 h, the materials had the most favorable properties. During this process, structures were obtained without defects (interruption of the wall), as well as with the most favorable surfaces for both the inside and outside. The dependencies were similar for all three analyzed polyurethanes.

As mentioned above, the three analyzed polymers differ in terms of Shore hardness. According to this scale, the C75A polymer is a soft polymer while C45D and C75D are hard ones. This is in line with the results where C75A materials were the most flexible and C75D materials were the stiffest.

When analyzing the surface morphology of the obtained materials, a set of parameters was selected: 0:100 water/ethanol, 20% PU concentration, and 24 h process time. After analyzing the mechanical properties of the materials from the three tested PU materials, it was decided that PU C45 D would be used for tests with a porogen.

The addition of a porogen met the expectations—the surface porosity was increased by about 9%. The average pore diameter also increased. However, there was a deterioration in the outer surface morphology; it was more folded and had many more surface irregularities. The washed-out porogen produced larger voids, resulting in a more uneven wall thickness. The wall thickness of the obtained structures was similar to that found in literature reports [9]. The use of pre-mixed polymer and the porogen can often result in irregular pore shapes while retaining residual porogen in the structure [65]. Therefore, by changing the type of porogen (its size), the pore size can be controlled [64,66]. In the studies presented here, small porogen residues can be seen in the obtained materials, hence we observed stiffening (changes of mechanical parameters) of the structure and an increasing wall thickness. Ahmed et al. [67] showed that porogen addition (NaHCO_3_) stabilized polyurethane cylindrical structures obtained via phase inversion, with water as a nonsolvent.

Vascular prostheses must have adequate mechanical properties to withstand blood pressure. In addition, they should be resistant to deformation and compression and have sufficient tensile strength to resist tensile loads when implanted into the body [68]. Our study showed that scaffolds with the addition of porogen were stiffer and less flexible than those without it. PVA addition lowered the mechanical properties of the materials. This result is not surprising since the greater the material’s porosity, the lower the scaffold’s mechanical properties [69]. The greater variability between samples and the higher standard deviations were due to the materials’ pore sizes and the porosity’s random nature. One of the most important mechanical parameters to be remembered when constructing vascular prostheses is the Young modulus [70]. The Young’s modulus for natural arteries is in the range of approx. 1.0–3.4 MPa [70,71], which is comparable to the PU C45D material. The reduction in tensile strength of the material with porogen corresponds with the work of Ahmed et al. [67]. The presence of macrovoids can reduce the mechanical properties of the materials [72].

Fibroblasts and endothelial cells are the most commonly used for cytotoxicity tests, and the ISO standard suggests L929 cells for these tests [73]. MTT analysis confirmed the lack of cytotoxic effects on cells of the material made of three examined polymers and the material obtained with 10% PVA. This was an entirely expected result. The PU used for preparation of the scaffolds was of medical grade. In the materials’ pores, no solvent or porogen remained, which would have leaked into the extract, giving a negative result.

Cylindrical scaffolds obtained using the phase inversion technique tend to be a promising material in testing for use as an artificial blood vessel. The next stage of research related to the obtained scaffolds is the analysis of material-blood interactions and the adhesion of human endothelial cells.

## 4. Materials and Methods

### 4.1. Materials

Polyurethane, ChronoFlex C75A, C45D and C75D, was bought in the form of pellets, from (AdvanSource Biomaterial, Wilmington, MA, U.SA). *N*,*N*-dimethylacetamide (DMAC), sodium chloride (NaCl, 99%), polyvinyl alcohol (PVA), were purchased from (Sigma Aldrich, Poznań, Poland). Hexane and ethanol (EtOH) were purchased from (POCH, Gliwice, Poland).

### 4.2. Preparation of Polyurethane Scaffolds—Selection of Process Parameters

**Figure 12:**
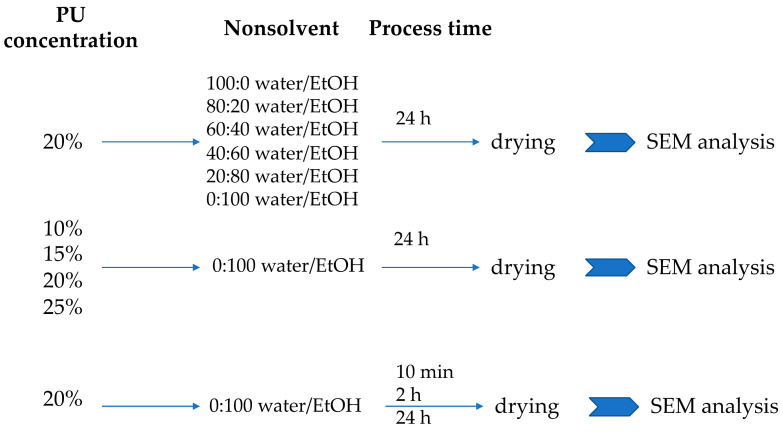
The scheme of testing selected process parameters.

The research was carried out in three parts for each type of tested polyurethane. First, the variable was nonsolvent concentration, second—the PU concentration, and third—the time of the process. Three types of polyurethane were used each time (Figure 12).

**Figure 13:**
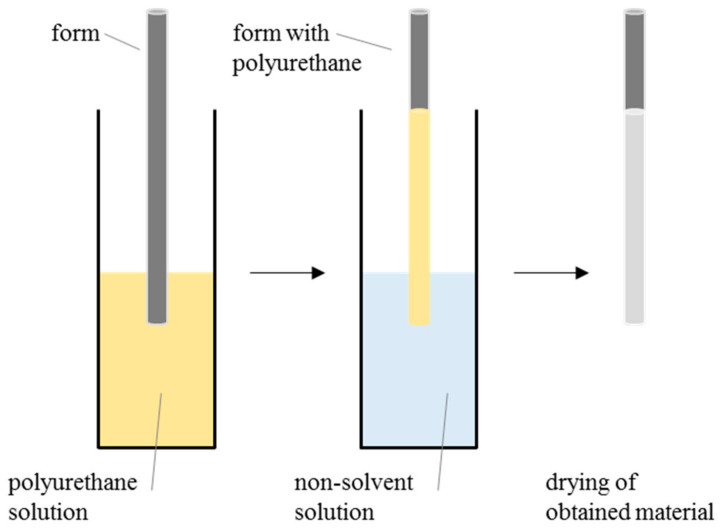
Scheme presenting the method for prosthesis casting.

Three types of PUs that differed in hardness were analyzed: C75A, C45D and C75D. Polyurethane pellets were washed with 70% EtOH/water solution, dried to constant weight at 40 °C and dissolved in DMAC to a given concentration. Four concentrations of PU solutions were examined: 10% (*w*/*v*), 15% (*w*/*v*), 20% (*w*/*v*) and 25% (*w*/*v*). Afterward, the stainless-steel matrix (with 6 mm diameter) was dipped in a PU solution and then immersed in a nonsolvent solution for a given time. Six nonsolvent solution differ in water/EtOH ratios were analyzed: 100:0 water/EtOH, 20:80 water/EtOH, 40:60 water/EtOH, 60:40 water/EtOH, 80:20 water/EtOH and 100:0 water/EtOH. Three process times were examined: 10 min, 2 h and 24 h. The resulting samples were removed from the nonsolvent solution, taken off the metal matrix, and allowed to dry at room temperature (RT) at high humidity. The scheme of the manufacturing process is presented in Figure 13.

### 4.3. Porogen Addition

In order to increase the number and size of surface pores, prostheses with the addition of a porogen were fabricated. Three porogens, namely NaCl (5% *w*/*v*, 10% *w*/*v*), PVA (5% *w*/*v*, 10% *w*/*v*) and hexane (5% *v*/*v*, 10% *v*/*v*) were selected. Porogen was added in given concentrations to the polymer solution. After porogen addition, the polymer solution was thoroughly mixed to distribute the porogen evenly throughout the polyurethane volume. Then, the prostheses were manufactured as described above.

### 4.4. Surface Characterization

The morphology of the obtained structures was examined with a scanning electron microscope (SEM, Phenom G1, Phenom World, Eindhoven, The Netherlands). Rectangular fragments were cut out from each cylindrical structure (*n* = 4). The internal and external surfaces were analyzed. Additionally, surface pore sizes were measured manually (*n* = 100 per variant) and wall thicknesses (*n* = 5, in 3 different spots) for materials considered to be most advantageous in terms of morphology. The measurements were performed based on SEM images with Fiji software [74].

For selected the materials, the surfaces and cross-sections were analyzed with a stereoscopic microscope (Leica, Wetzlar, Germany). The internal diameter was measurement manually using ImageJ.

### 4.5. Mechanical Testing

Mechanical properties were tested for the selected materials. Cylindrical structures (4 mm inner diameter, 60 mm length; *n* = 5) were subjected to a uniaxial stretching test according to protocols based on ASTM standards (D 638-02a and 882-02). The analyses were carried out using an Instron 3345 model with 5 mm∙min^−1^ head speed at RT and ambient humidity.

### 4.6. Porosity

Material porosity was determined using the gravimetric method [51,67]. The material porosity was calculated on the basis of its apparent density (ρ_app_) and known density of the polymer (ρ_p_ = 1.2 g/cm^3^ [75]), according to the following formula:

porosity (%) = (1 − ρ_app_/ρ_p_) × 100% (1)

where values of ρ_app_ were measured from the dimensions and weights of the materials (*n* = 5).

### 4.7. Cytotoxicity Evaluation

Cytotoxicity testing, MTT cell proliferation assay (Thiazolyl Blue Tetrazolium Bromide, Sigma-Aldrich, Poznań, Poland), was performed according to ISO 10993-5 [41]. L929 cells were cultured with Dulbecco’s Modified Eagle Medium (DMEM, ThermoFisher, Waltham, MA, USA) supplemented with bovine serum (10% *v*/*v*, ThermoFisher) and a mixture of penicillin–streptomycin antibiotics (1% *v*/*v*, ThermoFisher) in an incubator (37 °C, 5% CO_2_).

On the first day, cells were seeded at a density of 1·10^4^/per well and were grown for 24 h. Sterilized materials (using 70% ethanol, washed three times with sterile PBS) (*n* = 5) were incubated in DMEM medium for 24 h. After this time, material extracts were added to the cells and incubated for another 24 h. The negative control was cells with DMEM medium, and the positive control was cells treated with 0.1% Triton X. After removing the extracts from the wells, MTT solution (1 mg/mL DMEM, ThermoFisher, Waltham, MA, USA) was added. The plates were placed in an incubator for 4 h. After this time, the solution was removed and isopropanol was added to dissolve the formazan crystals. Absorbance was measured at a wavelength of 570 nm.

Cell viability was calculated using the following formula:

cell viability (%) = A_S_/A_C_ × 100% (2)

where A_S_ is the mean absorbance value of the sample and A_C_ is the mean absorbance value of the negative control. Cell viabilities are presented against negative control (which is assumed to be 100%).

### 4.8. Statistical Analysis

The results of the surface pore diameters, wall thicknesses, porosity, mechanical testing and cell viabilities were expressed as means ± SD. Statistically significant differences were investigated with a single-factor analysis of variance (ANOVA) for *p* < 0.05 with a post hoc Tukey’s test (OriginPRO 2020b, OriginLab Corporation, Northampton, MA, USA).

## 5. Conclusions

In conclusion, the influence of individual process parameters on the morphologies of the obtained scaffolds was demonstrated. It has been emphasized that any process parameter is significant in scaffold production using the phase inversion method. The study allowed selecting parameters, leading to the obtaining of cylindrical scaffolds with the most favorable morphologies and mechanical properties for use as vascular prostheses. The most advantageous porogen was also selected. For the selected process conditions, an analysis of mechanical properties was carried out, and the structures obtained with and without the addition of a porogen were compared. It was shown that the addition of PVA increased the range of pore diameters, average pore sizes, and the total porosity of the scaffold. At the same time, the materials with the porogen were characterized by a significantly higher Young’s modulus and a lower tensile strength. In addition, the structures produced by the proposed method, and with the selected parameters, were biocompatible, both with and without the addition of a porogen. The presented technology and materials can be employed for future vascular prosthesis manufacturing for medical trials and applications.

## Figures and Tables

**Table 1:** Symbolic description of prostheses obtained from different nonsolvent solutions (*n* = 3).

		100:0 Water/EtOH	80:20 Water/EtOH	60:40 Water/EtOH	40:60 Water/EtOH	20:80 Water/EtOH	0:100 Water/EtOH
C75A	(i)	+	+	+	‒	+	+
	(ii)	±	±	±		+	+
C45D	(i)	‒	‒	±	±	±	+
	(ii)	±	±	±	+	+	+
C75D	(i)	±	+	+	±	±	+
	(ii)	‒	–	±	±	±	±

(i) Uniform inner surface with surface porosity and (ii) stiffness/elasticity assessed manually; + meets the given criteria; ± does not fully meet the given criteria; − does not meet the given criteria.

**Table 2:** Symbolic description of prostheses obtained for various concentrations of PU (*n* = 3).

		10% PU	15% PU	20% PU	25% PU
C75A	(i)	‒	±	+	+
	(ii)	‒	±	+	+
C45D	(i)	‒	‒	+	+
	(ii)	‒		+	+
C75D	(i)	±	±	+	±
	(ii)	‒	±	±	±

(i) Uniform inner surface with surface porosity and (ii) stiffness/elasticity assessed manually; + meets the given criterion; ± does not fully meet the given criterion; ‒ does not meet the given criterion.

**Table 3:** Symbolic description of prostheses obtained for various times of the process (*n* = 3).

		10 min	2 h	24 h
C75A	(i)	−	−	+
	(ii)	±	±	+
C45D	(i)	±	+	+
	(ii)	±	±	+
C75D	(i)	+	+	+
	(ii)	±	±	+

(i) uniform inner surface with surface porosity and (ii) stiffness/elasticity assessed manually; + meets the given criterion; ± does not fully meet the given criterion; − does not meet the given criterion.

**Table 4:** Mechanical properties of C75A, C45D and C75D materials obtained with selected process parameters and cytotoxicity results (*n* = 5).

Polymer Type	C75A	C45D	C75D
Polymer concentration (%)	20		
Nonsolvent	0:100 water/EtOH		
Process time (h)	24 h		
Young’s modulus (MPa)	*		
	2.4 ± 0.1	3.6 ± 1.5	38.5 ± 4.3
Tensile strength (MPa)	*		
	9.8 ± 2.5	11.2 ± 1.2	4.1 ± 0.9
Elongation at break [mm/mm]	*		
	7.8 ± 0.9	4.7 ± 0.4	0.8 ± 0.3
Cell viability (%)	87 ± 4	92 ± 4	89 ± 4

* *p* < 0.05.

**Table 5:** Symbolic description of prostheses obtained for various porogens (*n* = 3).

	5% NaCl	10% NaCl	5% PVA	10% PVA	5% Hexane	10% Hexane
(i)	+	+	+	+	+	+
(ii)	±	±	±	+	+	±

(i) uniform inner surface with surface porosity, (ii) stiffness/elasticity assessed manually; + meets the given criterion; ± does not fully meet the given criterion.

**Table 6:** Comparison of basic physical and mechanical parameters of scaffolds obtained for selected process parameters (*n* = 5).

	PU C45D	PU C45D_10%PVA	
Polymer type	C45D		
Polymer concentration (%)	20		
Nonsolvent	0:100 water/EtOH		
Process time (h)	24		
Porogen	–	10% PVA	
Wall thickness (μm)	202.0 ± 19.0	*	278.0 ± 78.0
Internal diameter (mm)	5.35 ± 0.1		5.41 ± 0.2
Average surface pore diameter (μm)	9.6 ± 3.2	*	15.2 ± 6.4
Min surface pore diameter (μm)	3.3		5.5
Max surface pore diameter (μm)	20.4		41.2
Porosity (%)	59 ± 2	*	68 ± 3
Young’s modulus (MPa)	3.6 ± 1.5		9.7 ± 4.3
Tensile strength (MPa)	11.2 ± 1.2	*	6.9 ± 2.3
Elongation at break [mm/mm]	4.7 ± 0.4	*	1.9 ± 0.2
Cell viability (%)	92 ± 4		86 ± 9

* *p* < 0.05.

## Data Availability

Data is contained withinh the article.
